# Intralymphatic GAD-Alum (Diamyd®) Improves Glycemic Control in Type 1 Diabetes With HLA DR3-DQ2

**DOI:** 10.1210/clinem/dgac343

**Published:** 2022-06-06

**Authors:** Christoph Nowak, Marcus Lind, Zdenek Sumnik, Terezie Pelikanova, Lía Nattero-Chavez, Elena Lundberg, Itxaso Rica, Maria A Martínez-Brocca, MariSol Ruiz de Adana, Jeanette Wahlberg, Ragnar Hanas, Cristina Hernandez, Maria Clemente-León, Ana Gómez-Gila, Marta Ferrer Lozano, Theo Sas, Stepanka Pruhova, Fabricia Dietrich, Sara Puente-Marin, Ulf Hannelius, Rosaura Casas, Johnny Ludvigsson

**Affiliations:** Department of Neurobiology, Care Sciences and Society, Karolinska Institutet, 14183 Huddinge, Sweden; Diamyd Medical AB, 11135 Stockholm, Sweden; Department of Molecular and Clinical Medicine, University of Gothenburg, 41345 Gothenburg, Sweden, Sahlgrenska University Hospital, Gothenburg and NU-Hospital Group, S41553, Uddevalla, Sweden; Department of Pediatrics, 2nd Faculty of Medicine, Charles University and Motol University Hospital, 15000 Prague, Czech Republic; Diabetes Centre of the Institute of Clinical and Experimental Medicine, 14000 Prague, Czech Republic; Department of Endocrinology and Nutrition, Hospital Universitario Ramón y Cajal, 28034 Madrid, Spain; Institution of Clinical Science, Department of Pediatrics, Umeå University, Norrland University Hospital, 93451 Umeå, Sweden; Department of Pediatric Endocrinology, Cruces University Hospital, 48902 Bilbao, Ciberdem, Spain; Department of Endocrinology, Virgen Macarena Hospital, Department of Endocrinology and Nutrition, Virgen Macarena University Hospital, 41009 Sevilla, Spain; Diabetes Unit, Department of Endocrinology and Nutrition, Ibima, Ciberdem, General University Hospital, 29010 Malaga, Spain; Department of Endocrinology in Linköping and Department of Health, Medicine and Caring Sciences, Linköping University, 58183 Linköping, Sweden; Department of Internal Medicine, School of Health and Medical Sciences, Örebro University, 70281 Örebro, Sweden; Department of Pediatrics, NU Hospital Group, 45153 Uddevalla, Sweden; Department of Endocrinology and Nutrition, Vall d’Hebron Hospital, 08035 Barcelona, Ciberdem, Spain; Department of Endocrinology, Pediatric Service, Vall d’Hebron Hospital, 08035 Barcelona, CibererSpain; Pediatric Endocrinology Service, Virgen del Rocío University Hospital, 41013 Sevilla, Spain; Department of Pediatric Endocrinology, Miguel Servet University Hospital, 50009 Zaragoza, Spain; Diabeter, National Treatment and Research Center for Children, Adolescents and Young Adults with type 1 diabetes, and Department of Pediatric Endocrinology, Erasmus University Medical Center, 3015 Rotterdam, The Netherlands; Department of Pediatrics, 2nd Faculty of Medicine, Charles University and Motol University Hospital, 15000 Prague, Czech Republic; Division of Pediatrics, Department of Biomedical and Clinical Sciences, Faculty of Medicine and Health Sciences, Linköping University, 58183 Linköping, Sweden; Division of Pediatrics, Department of Biomedical and Clinical Sciences, Faculty of Medicine and Health Sciences, Linköping University, 58183 Linköping, Sweden; Diamyd Medical AB, 11135 Stockholm, Sweden; Division of Pediatrics, Department of Biomedical and Clinical Sciences, Faculty of Medicine and Health Sciences and Crown Princess Victoria Children´s Hospital, Linköping University, 58183 Linköping, Sweden; Division of Pediatrics, Department of Biomedical and Clinical Sciences, Faculty of Medicine and Health Sciences and Crown Princess Victoria Children´s Hospital, Linköping University, 58183 Linköping, Sweden

**Keywords:** type 1 diabetes, GAD-alum, GAD65, Diamyd, HLA DR3-DQ2, continuous glucose monitoring, C-peptide, HbA1c, antigen-specific immune therapy

## Abstract

**Aims:**

Residual beta cell function in type 1 diabetes (T1D) is associated with lower risk of complications. Autoantigen therapy with GAD-alum (Diamyd) given in 3 intralymphatic injections with oral vitamin D has shown promising results in persons with T1D carrying the human leukocyte antigen (HLA) DR3-DQ2 haplotype in the phase 2b trial DIAGNODE-2. We aimed to explore the efficacy of intralymphatic GAD-alum on blood glucose recorded by continuous glucose monitoring (CGM).

**Methods:**

DIAGNODE-2 (NCT03345004) was a multicenter, randomized, placebo-controlled, double-blind trial of 109 recent-onset T1D patients aged 12 to 24 years with GAD65 antibodies and fasting C-peptide > 0.12 nmol/L, which randomized patients to 3 intralymphatic injections of 4 μg GAD-alum and oral vitamin D, or placebo. We report results for exploratory endpoints assessed by 14-day CGM at months 0, 6, and 15. Treatment arms were compared by mixed-effects models for repeated measures adjusting for baseline values.

**Results:**

We included 98 patients with CGM recordings of sufficient quality (DR3-DQ2-positive patients: 27 GAD-alum-treated and 15 placebo-treated). In DR3-DQ2-positive patients, percent of time in range (TIR, 3.9-10 mmol/L) declined less between baseline and month 15 in GAD-alum-treated compared with placebo-treated patients (-5.1% and -16.7%, respectively; *P* = 0.0075), with reduced time > 13.9 mmol/L (*P* = 0.0036), and significant benefits on the glucose management indicator (*P* = 0.0025). No differences were detected for hypoglycemia. GAD-alum compared to placebo lowered the increase in glycemic variability (standard deviation) observed in both groups (*P* = 0.0219). Change in C-peptide was correlated with the change in TIR.

**Conclusions:**

Intralymphatic GAD-alum improves glycemic control in recently diagnosed T1D patients carrying HLA DR3-DQ2.

More than 100 years after the discovery of insulin, individuals with type 1 diabetes (T1D) still lack access to a treatment that halts disease progression and protects functional beta cells to prevent serious complications and early death (1). Maintaining even a minimum residual beta-cell function improves glycemic control and reduces the risk of severe hypoglycemia and retinopathy (2-4). Although several investigational drugs have shown significant preservation of C-peptide (a surrogate for endogenously produced insulin) in recent-onset T1D, direct effects on disease complications have never been shown as such events are rare in the first years after diagnosis in patients receiving standard of care treatment. Glycated hemoglobin (HbA1c) is associated with diabetes complications including cardiovascular disease and mortality (5-7) and is an accepted surrogate endpoint in clinical trials. Although C-peptide preservation has been associated with improved HbA1c (8), beta cell-preserving interventions have yet to show direct treatment benefits on HbA1c as well. However, HbA1c is a blunt measure of glycemic control because it lacks information on acute glycemic excursions as well as blood glucose variability (9, 10), and a more comprehensive and clinically meaningful measure of glucose balance in clinical studies of recent onset T1D has become possible by using glucose sensors.

Continuous glucose monitoring (CGM) tracks blood glucose levels throughout day and night by performing glucose measurements at regular intervals such as every 5 minutes, providing dynamic data on intra- and inter-day variability. CGM recordings over a continuous period capture important information on diabetes control that is not captured by patients’ capillary blood glucose measurements, which are only carried out a few times per day; or by HbA1c, which quantifies the average blood glucose over the preceding 3 months. Both the American Diabetes Association (11) and an international consensus (12) recommend the use of time in range (time in range [TIR], 3.9-10.0 mmol/L or 70-180 mg/dL) as well as other CGM-derived parameters such as time above/below range and glycemic variability to be used in clinical care of diabetes. TIR is associated with HbA1c (13, 14) and diabetic complications (15, 16). CGM also measures glycemic variability, which is an independent risk factor for diabetes complications and nocturnal hypoglycemia (17, 18).

It has been shown that individuals with the genetic human leukocyte antigen (HLA) haplotype DR3-DQ2 represent a specific endotype of T1D with a tendency to develop primary autoimmunity against glutamate decarboxylase 65 kDa (GAD65) (19). Data from the recently completed placebo-controlled phase 2b DIAGNODE-2 trial showed that those participants with T1D who carried the HLA DR3-DQ2 haplotype benefited significantly from 3 monthly intralymphatic administrations of recombinant human GAD65 (rhGAD65) in alum (GAD-alum/Diamyd) with regard to preserving insulin-producing capacity (20), confirming observations previously reported in a large-scale meta-analysis (21). The benefits of intralymphatic GAD-alum therapy on metabolic control were further supported by a significantly higher number of subjects in partial remission as indicated by insulin-adjusted HbA1c < 9 (20), but only a nonsignificant treatment benefit for lower HbA1c was seen.

Our objective was to explore the efficacy of 3 intralymphatic injections of GAD-alum (Diamyd) immunotherapy combined with oral vitamin D supplementation compared with placebo on blood glucose recorded by continuous glucose monitoring in children and young adults with recent-onset T1D enrolled in the 15-month DIAGNODE-2 trial.

## Materials and Methods

### DIAGNODE-2 Study

**Figure 1.**
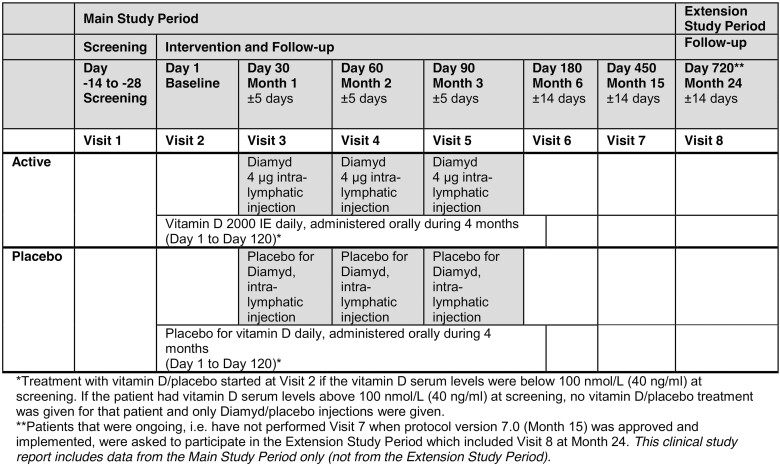
DIAGNODE-2 study visit schedule.

DIAGNODE-2 (NCT03345004) (20), a phase 2b randomized, double-blind, placebo-controlled trial in 109 persons aged 12 to 24 years with recent-onset T1D, detectable GAD65 antibodies, and fasting serum C-peptide > 0.12 nmol/L (>0.36 ng/mL) evaluated the efficacy of intralymphatic administration of three doses of 4 µg of GAD-alum with oral vitamin D supplementation compared with placebo in terms of preserving endogenous insulin secretion (standard mixed meal tolerance test [MMTT], stimulated C-peptide area under the curve between 0 and 120 minutes [AUC_0-120min_]). DIAGNODE-2 was performed at 18 diabetes clinics in the Czech Republic, the Netherlands, Spain, and Sweden. Screening was performed between December 2017 and April 2019. In the course of the DIAGNODE-2 trial, a meta-analysis of clinical data from 3 prior GAD-alum trials that evaluated subcutaneous administration of GAD-alum was published (21). This meta-analysis convincingly concluded that the effect of GAD-alum on beta-cell functional preservation depends on the patient’s HLA genotype, namely, the presence of the HLA DR3-DQ2 haplotype. In DIAGNODE-2, 46 patients carried this haplotype, 19 in the placebo group and 27 in the actively treated arm. Figure 1 illustrates the study visit schedule.

### Continuous Glucose Monitoring

In DIAGNODE-2, 14-day CGM recordings were obtained at the screening visit as well as the 6-month and 15-month (end-of-study) visits (Fig. 1). Readings were obtained with the FreeStyle Libre Pro FGM System, which is a professional CGM device used for retrieving masked CGM data and thereby avoiding affecting any ongoing standard-of-care glucose-lowering therapy. It is a very accurate system with an overall mean absolute relative difference of 12.3% compared with the recognized standard for laboratory analysis of blood glucose (Yellow Spring Instrument references, according to the manufacturer Abbott). We compared for individuals receiving active or placebo treatment the following: TIR (3.9-10.0 mmol/L), glucose variability (standard deviation) in mmol/L, time in hypoglycemia (<3.0 mmol/L), time in hyperglycemia (>13.9 mmol/L), the glucose management indicator (a measure to estimate HbA1c based on CGM mean glucose data calculated as 3.31 + 0.02392 × [mean glucose in mg/dL], and expressed as % DCCT units, similar to HbA1c) (22).

### C-peptide Measurement

Meal-stimulated C-peptide was assessed using a standardized MMTT. Participants were instructed to attend the MMTT fasted (>10 hours) and abstinent from any short-acting or direct-acting insulins (>6 hours). C-peptide during the 2-hour MMTT was measured at 5 time points and the AUC_0-120min_ determined using the trapezoidal rule.

### Statistical Analysis

After the exclusion of deficient datasets (<50% recorded data during the 14-day period), low-quality datasets (eg, an outlier with maximum recordings throughout the period), and technical failure (eg, where the sensor was not properly attached and hence did not record proper values), a total of 98 patients were included in the CGM analysis with a median (mean) CGM wear time of 14 (13) days. All included participants had > 90% recorded data during the 14-day period. There were 27 patients treated with Diamyd and 15 treated with placebo in the predefined subgroup of individuals carrying HLA DR3-DQ2. CGM raw data were processed using the *cgmanalysis* package in R version 4.0.4 (23). Change from baseline was analyzed with restricted maximum likelihood mixed models for repeated measures (REML-MMRM) adjusted for subject (random effect) and baseline value, visit, treatment, DR3 (fixed effects) and baseline*visit as well as treatment*visit*DR3 interactions. For the interpretation of the data in the context of other results from the DIAGNODE-2 study, it should be noted that the baseline value for CGM analysis corresponds to the screening visit, which took place 14 to 28 days before the study baseline visit, where baseline samples for all other outcomes (HbA1c, C-peptide etc.) were obtained. To assess the association between C-peptide and percent spent in TIR, we used scatterplots of the raw values and fitted nonparametric locally estimated scatterplot smoothing curves.

## Results

Among individuals carrying HLA DR3-DQ2, the 27 GAD-alum-treated individuals were of similar age and body stature as the 15 individuals treated with placebo, but there was a somewhat lower proportion of female subjects (33%) in the GAD-alum group compared with the placebo group (47%) (Table 1). Because individuals carrying HLA DR3-DQ2 have been identified as the responder population for GAD-alum treatment and will be enrolled in the planned confirmatory phase 3 trial DIAGNODE-3, the current study focuses on these individuals unless otherwise stated.

**Table 1.** Baseline characteristics (screening visit)

	Present DR3-DQ2		Absent DR3-DQ2	
	Diamyd	Placebo	Diamyd	Placebo
N	27	15	26	30
Female/male	9/18	7/8	10/16	18/12
Age, mean (SD), median, y	16.6 (4.0), 15	16.2 (3.8), 15	15.6 (3.5), 14.5	16.8 (4.8), 14
BMI	21.9 (3.7)	21.0 (3.2)	20.7 (2.9)	22.6 (4.9)
SBP	116 (11)	110 (18)	113 (9)	114 (13)
DBP	69 (8)	68 (9)	66 (9)	71 (10)
Height	169.9 (9.7)	166.9 (11.1)	169.1 (8.9)	167.2 (10.7)
Weight	63.4 (12.7)	58.7 (12.5)	59.8 (11.9)	64.2 (19.4)
Insulin dose, IU/kg body weight per day, Baseline	0.32 (0.19)	0.47 (0.29)	0.48 (0.27)	0.49 (0.36)
Insulin dose, IU/kg body weight per day, month 15	0.46 (0.22)	0.65 (0.35)	0.60 (0.30)	0.50 (0.37)

Abbreviations: BMI, body mass index; DBP, diastolic blood pressure; SBP, systolic blood pressure, SD, standard deviation.

**Figure 2.**
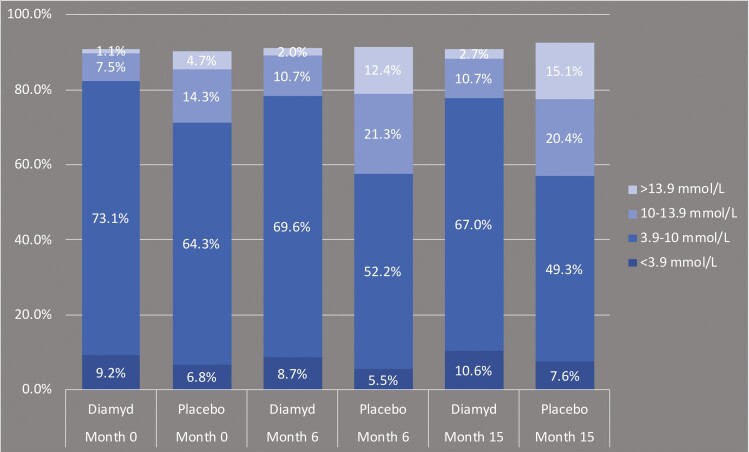
Mean percent of time during the 14-day CGM recording periods spent in different glycemic ranges (individuals with HLA DR3-DQ2).

**Figure 3.**
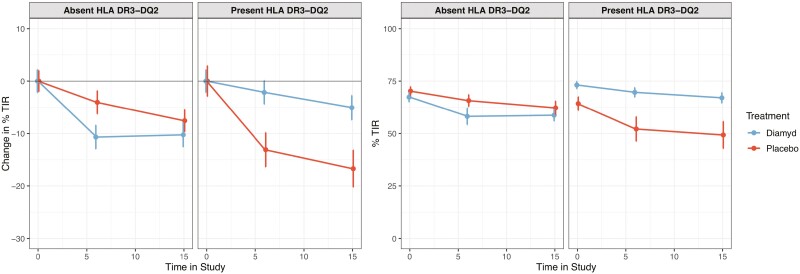
Change in percent of time in range (3.9-10 mmol/L) during the study. The left panel shows the MMRM-predicted change where the difference between groups in the HLA DR3-DQ2 population reached statistical significance at months 6 (*P* = 0.0072) and 15 (*P* = 0.0075). The right shows the original data. Error bars represent standard errors.

Figure 2 shows the mean percentage of time during the 14-day recording that each treatment group with responder individuals carrying HLA DR3-DQ2 spent in different glycemic ranges. During the 15-month study period, the mean percentage of time spent within the time in range (TIR, 3.9-10 mmol/L) remained fairly stable in patients treated with GAD-alum, whereas there was a marked decline of the TIR in the placebo-treated group (from 64.3% at screening to 49.3% at month 15). This was driven by an increase in the amount of time spent in the moderate and more pronounced hyperglycemic ranges of 10-13.9 mmol/L and > 13.9 mmol/L, respectively. In MMRM analysis, there was a significant benefit for active treatment over placebo for change from baseline in TIR, both at 6 months (*P* = 0.0072) and 15 months (*P* = 0.0075) in individuals with HLA DR3-DQ2, whereas there was no difference in individuals lacking DR3-DQ2 (Fig. 3).

There was a nominally significant benefit for GAD-alum treatment compared with placebo in lowering the increase in the standard deviation of blood glucose readings during the study period (*P* = 0.0420 at 6 months, and *P* = 0.0219 at 15 months; Suppl Fig 1 (24)), but there was no significant difference between the treatment arms regarding the coefficient of variation (standard deviation divided by the mean) measure of glycemic variability (Suppl Fig 2 (24)).

No differences between the treatment arms were found for change from baseline in time in the hypoglycemic range below 3 mmol/L (<54 mg/dL), as shown in Suppl Fig 3 (24).

GAD-alum treatment showed significant benefit over placebo in limiting the time with very high glucose levels above 13.9 mmol/L (>250 mg/dL, Suppl Fig 4 (24)). There was also a nominally significant treatment benefit on the glucose management indicator (Suppl Fig 5 (24)) indicating a low and stable mean glucose level during the study in the GAD-alum-treated patients with HLA DR3-DQ2 in contrast to an increase in mean glucose values and variation in placebo-treated patients with DR3-DQ2. We found a strong positive correlation between HbA1c and the glucose management indicator (“estimated HbA1c”) based on mean CGM glucose readings, particularly at the 6-month and 15-month visits (Suppl Fig 6 (24)).

**Figure 4.**
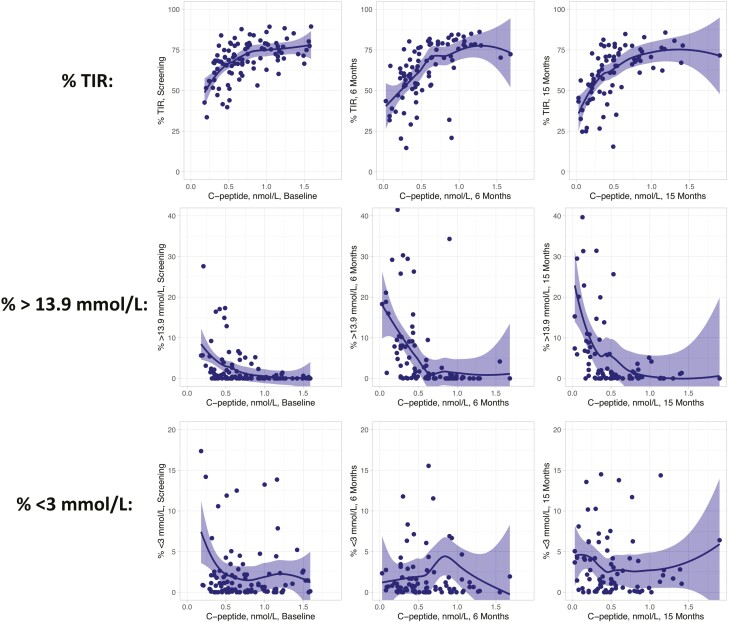
Scatterplots showing the association between AUC_0-120min_ C-peptide (raw values in nmol/L) on the x-axis and percent spent in time in range (upper), >13.9 mmol/L (middle), and < 3 mmol/L (lower panel) on the y-axis. A locally estimated scatterplot smoothing regression line with 95% confidence interval has been fitted. All patients with CGM data are included, regardless of treatment condition.

**Figure 5.**
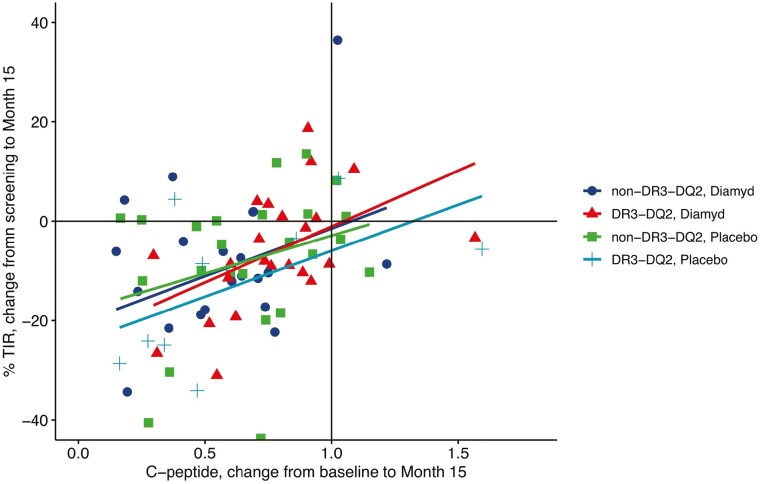
Scatterplot showing the association between change in AUC_0-120min_ C-peptide (ratio between values at month 15 divided by baseline values in nmol/L) on the x-axis and change in time in range (difference between screening visit and month 15 in percent). Simple linear regression lines have been fitted. The figure suggests a positive correlation between preservation of C-peptide and reduced decline in TIR during the study period.

To assess the association between residual insulin secretion and metabolic outcomes, we prepared scatterplots between AUC_0-120min_ C-peptide values and percent of time spent in TIR, <3 mmol/L, or > 13.9 mmol/L at different time points in the study (Fig. 4). For each of the assessments, the associations for TIR appeared to divide into 2 parts. Above a threshold of about 0.6 nmol/L of stimulated C-peptide level, the association was essentially flat, whereas below 0.6 nmol/L, there was a positive correlation with a steep slope. A similar threshold effect around 0.6 nmol/L was apparent for hyperglycemia, where C-peptide values below 0.6 nmol/L correlated with increasingly more time spent above 13.9 mmol/L in blood glucose. For hypoglycemia (<3 mmol/L), this association was less apparent. Similarly, the change in C-peptide during the study was positively correlated with the change in TIR across treatment groups and HLA DR3-DQ2 presence or absence (Fig. 5), with an average Pearson correlation of *r* = 0.40 (*P* = 0.0003).

## Discussion

In exploratory studies of CGM data obtained in a placebo-controlled phase 2b trial assessing intralymphatic GAD-alum in recent-onset T1D, we found significant treatment benefits compared to placebo for TIR, hyperglycemia, glycemic variability as measured by the standard deviation, and mean glucose (assessed by the glucose management indicator) in the responder population of patients carrying HLA DR3-DQ2. To our knowledge, this is the first immune intervention trial in T1D to show treatment effects on glycemic control as indicated by CGM data. We also found suggestive positive correlations between higher C-peptide values and improved TIR, as well as between a larger decline in C-peptide and larger fall in TIR during the study.

It is well known from several studies that residual beta-cell function assessed by C-peptide is associated with a reduction in acute and late complications (3, 4, 8, 25), and reduced mortality (26). However, it has been difficult in previous immune intervention trials at the onset of T1D to see an effect on metabolic control—even in trials that show efficacy for preservation of residual beta-cell function. This might be due to the general diabetes management policy to aim for as good metabolic control as possible with low and stable HbA1c, which can be reached with modern standard-of-care treatment in both, an actively treated group and a placebo-treated group. It is also possible that HbA1c might be too blunt a measure of glycemic control to show significant effects in trial sizes typical for immune interventions, although well-powered phase 3 trials might be able to detect treatment benefits.

In the DIAGNODE-2 study, we found significant treatment effects on C-peptide preservation and positive trends suggestive of benefits on glycemic control in patients with HLA DR3-DQ2 (20). Here, we also show statistically and clinically significant improvements in blood glucose fluctuations. Patients with HLA DR3-DQ2 who received GAD-alum treatment spent significantly more time in the glycemic target range and less time with very high blood glucose (>13.9 mmol/L) already after 6 months and more so after 15 months than those patients who received placebo. The significant treatment effect seen on glycemic control and its correlation with the previously observed effects on the preservation of C-peptide further supports the clinical benefit of GAD-alum treatment as well as the legitimacy of the HLA DR3-DQ2 subgroup. Consequently, only persons with T1D carrying HLA DR3-DQ2 will be enrolled in the planned confirmatory phase 3 trial DIAGNODE-3 of intralymphatic GAD-alum.

Changes in TIR have previously been associated with changes in HbA1c, where a 10% increase in TIR was associated with an approximately 5 mmol/mol higher HbA1c (12, 15, 16). As would be expected, an association between TIR and HbA1c was also seen in DIAGNODE-2. The estimated treatment effect on TIR for patients with HLA DR3-DQ2 was approximately 10% (Fig. 3), and the estimated treatment effect on HbA1c was approximately 5 mmol/mol (20), suggesting that the indicated effect on HbA1c is indeed true.

The clinical effects on metabolic outcomes are most likely a consequence of the preservation of beta cell function. This is supported by the correlation between TIR and C-peptide at all three assessment time points, with a steep and distinct association for stimulated C-peptide values below 0.6 nmol/L, although above this threshold, the effects of higher C-peptide on metabolic control appear to be more limited. These results support the benefits of preserved residual insulin production to uphold glycemic control. This suggests that a stimulated C-peptide of at least 0.6 nmol/L may be an important treatment target and that the correlation of effects on C-peptide and glycemic control in clinical trials may vary depending on the baseline status of the study sample. We also found significant correlations between C-peptide and glycemic variability as measured by the coefficient of variation (Pearson correlations between –0.60 and –0.45 for the different assessment points).

Current CGM devices may not be sufficiently reliable for detecting hypoglycemia in routine use outside of clinical trials (27, 28). In the current study, no difference between treatment arms in time spent in the hypoglycemic range was detected, which might be due to absent treatment effect but could also be related to possibly lower accuracy of the CGM devices in the hypoglycemic range as well as the generally well-controlled diabetes (< 4% of the time spent below 3 mmol/L across groups) as all participants received standard-of-care treatment. The continuous nature of a 14-day CGM recording within a trial context, however, provides clinically relevant summary data and a unique opportunity to capture blood glucose variability at night and at times where traditional blood glucose readings would not be done. CGM data therefore add important supportive insights into glycemic control that are not captured by HbA1c, which only provides an average measure of blood glucose over the preceding 3 months. TIR based on CGM has already been shown to correlate with occurrence of diabetic complications (13, 14), and further clinical research will tell if endpoints based on CMG data correlate equally well or better than HbA1c with clinical outcomes. CGM as an endpoint was recently discussed during a Critical Path Institute workshop (15-16 June 2021) (29). Although speakers from the European Medicines Agency and the Food and Drug Administration emphasized that more evidence was needed to establish CGM as a clinically validated endpoint, patient advocates supported the relevance of CGM results as clinically relevant indicators of disease management. In particular, glycemic variability was singled out as relevant for patients, with 1 affected person describing it as “less variability counts” meaning fewer lows and fewer highs in blood sugar and “less of that rollercoaster” of insulin dose adjustments and fear of nocturnal blood sugar spikes or mistakes in calibrating one’s insulin doses.

### Limitations

This is an exploratory analysis considered to be hypothesis-generating rather than confirmatory; hence, *P* values are not adjusted for multiple testing. There were some imbalances between the groups at baseline regarding demographics and a lower % TIR in the placebo group.

### Summary

Exploratory analyses of 14-day CGM recordings at three time points in the phase 2b trial DIAGNODE-2 demonstrate that intralymphatic GAD-alum compared with placebo treatment in recent-onset T1D patients with HLA DR3-DQ2 has significant benefits for time in the glycemic target range (3.9-10 mmol/L; 70-180 mg/dL), glycemic variability as measured by the standard deviation, and reduced time with very high glucose levels, whereas no differences were found between the groups regarding time in the hypoglycemic range. This clinical efficacy is likely related to preservation of beta-cell function.

## Data Availability

Data are not available by open access since participants did not consent to making their data publicly available in that way. Data underlying the current manuscript will be made available after reasonable request via a data transfer agreement to those interested in collaborations on further research. Such requests should be addressed to the corresponding author. Clinical Trial Registration number: NCT03345004

## Abbreviations

AUC_0-120min_: area under the curve between 0 and 120 minutes
CGM: continuous glucose monitoring
GAD: glutamic acid decarboxylase
HbA1c: glycated hemoglobin
HLA: human leukocyte antigen
MMTT: mixed meal tolerance test
T1D: type 1 diabetes
TIR: time in range
